# Efficient removal of methylene blue using Azolla/biochar composite: adsorption behavior and post-use valorization for methanol oxidation

**DOI:** 10.1038/s41598-026-60258-7

**Published:** 2026-07-15

**Authors:** Omnia M. Salem, Noha Abelwahab, Fatma Mohamed

**Affiliations:** 1 Chemistry Department, Faculty of Science, Beni-Suef University, Beni-Suef, 62514 Egypt; 2 Nanophotonics and Applications (NPA) Lab, Faculty of Science, Beni-Suef University, Beni-Suef, 62514 Egypt; 3 Materials Science Research Lab, Chemistry Department, Faculty of Science, Beni-Suef University, Beni-Suef, 62514 Egypt; 4 Department of Microbiology, Water and Environment Research Institute (SWERI), Agricultural Research center (ARC), Soil, Giza, 12619 Egypt

**Keywords:** Azolla, Biochar, Biomaterial, Adsorption, Methylene blue, Sustainability, DMFCs, Chemistry, Engineering, Environmental sciences, Materials science

## Abstract

The use of green and sustainable chemistry offers an efficient approach for synthesized innovative materials to address wastewater treatment challenges with declined energy consumption. This study addressed the significant global issues of water pollution from industrialization and population growth by designing cost effective, high-capacity materials utilizing for the removal of Methylene blue dye (MB). A novel bio-composite material (AZ/BC) was synthesized by amalgamating Azolla pinnata extract and biochar by ultrasonication technique. The resultant biomaterials were evaluated using techniques such as XRD, FTIR, and SEM to examine its surface appearance and crystalline structure. Batch adsorption experiments were conducted determine optimal conditions for MB removal, while Monte Carlo simulations were validated the molecules interaction between MB and the AZ/BC surface. The synthesized AZ/BC biomaterial exhibited a successful structural transition to uniformly stacked layers with high porosity, attaining a maximum methylene blue removal effectiveness of 96.21% within 120 min. Adsorption kinetics followed a pseudo-second-order model, and the Langmuir isothermal best described the monolayer adsorption process. Additionally, the used adsorbent was effectively repurposed as an efficient electrocatalyst for methanol oxidation in fuel cells, demonstrating excellent stability and highlighting a sustainable approach for energy production from wastewater treatment material.

## Introduction

Water is clearly one of the single most important natural resources, vital for the preservation of life on our planet. It represents the fundamental basis for biological, Chemical, and other physical properties^1^. In addition, water constitutes one of the 17 sustainable development goals (SDGs) established for 2030 worldwide and is regarded as a fundamental human right. Nowadays, water contamination is receiving worldwide attention as a critical environmental issue, largely because of the apparent shortage of water. Especially industrial development, climate change, large scale urbanization, intensive agriculture, mining activities, pharmaceuticals, heavy metals into water resources, and the release of hazardous organic and inorganic compounds^2^.

Dye pollutant poses a significant risk to environmental ecosystems and human health owing to its persistence and resistance to degradation or adsorption^3^. Most dyes demonstrate nonbiodegradable and chemically stable properties, allowing them to persist in aquatic environments for prolonged durations^4^. When released into aquatic environments, these chemicals obstruct light penetration, thereby disrupting photosynthesis in aquatic vegetation. This decline in photosynthetic activity results in diminished dissolved oxygen levels, hence altering aquatic ecosystem equilibrium and adversely impacting the survival of organisms^5,6^. These detrimental effects can ultimately lead to the death of aquatic organisms and a substantial reduction in biodiversity. Exposure to dye-contaminated water is linked to several toxicological effects, including carcinogenicity, mutagenicity, allergic responses, dermatitis, skin irritation, respiratory problems, reproductive toxicity, and endocrine disruption^7–9^. Building upon these broader environmental and health concern, MB emerges as a representative example of a widely used dye with considerable environmental repercussions^10^. It’s extensively employed across many industrial sectors, particularly in textiles, where it adds heavily to color loaded effluent^11^. In addition to the textile industry and other sectors such as paper, leather, paint, and printing also play a considerable role in discharge into aquatic habitats^6,12^. It has been claimed that more than 100,000 commercially available dye compounds, such as MB are dumped into water bodies, leading to significant ecological and health associated repercussions^13^. The presence of MB not only produces apparent contamination, such decolorization and foul odors, Moreover, it prevents the penetration of light into water, thereby decreasing the photosynthetic activity of aquatic plants^14,15^. The imbalance of the environment results in severe harm to aquatic creatures, as dissolved oxygen levels decrease. Furthermore, it has a chemical stability and resistance to biodegradation^16^. In addition, preliminary studies have demonstrated that MB can be effectively removed from wastewater by utilizing different adsorbents, with removal efficiencies achieved up to 69.6% for using rough reed fibers and up to 99% in batch test using by activated carbon/natural zeolite (AC/NZ) composites at optimal conditions, demonstrating the effectiveness of advanced adsorbent materials in addressing MB contamination in industrial effluents^17,18^.

Numerous technologies have been implemented for the decontamination and purification of polluted water. Several of these procedures have limits due to the necessity of multiple processes, large sludge generation, the use of non-reusable chemical reagents, high costs, and intense electrical demand^19^. Among these approaches, adsorption is the preeminent chemical and physical methods for reducing environment contamination, particularly in the removal of heavy metal ions, as a result of its straightforward design, exceptional efficacy in impurity removal, lack of secondary pollution, cost effective, high separation selectivity, recyclability, minimal energy demand, ease of operation, environmental friendliness, and scalability^20^. The effective adsorption process necessitates an adsorbent to achieve optimal efficiency. Many studied have examined and adjusted the adsorbent to enhance the efficacy of the adsorption process^21^.

Biosorbents have gained significant importance in the development of sorbents for wastewater treatment^22^. These materials possess more analogous active sites than those present in ion exchange polymeric sorbents, based on their biological origin. The current objective was to employ the waste biomass Azolla sp. as an adsorbent. This analysis aims to facilitate a discussion regarding the variability in the sorption system and the improvement of system attributes^23^. Azolla is tiny and buoyant, yet it may proliferate extensively, forming extensive mats; it may also assimilate nitrogen from the atmosphere through symbiotic cyanobacteria^24^. It can endure in aquatic environments, with optimal development shown at temperatures ranging from 25 to 35 °C. This cultivar has progressed into a plant capable of rapidly colonizing regions and growing at an extraordinary rate, doubling its biomass every two to three days. Azolla demonstrates significant capacity to absorb heavy metals and contaminants from sewage water, and it is also employed as feed for fish, fowl, and cattle^25^.

Azolla is recognized as a valuable super plant because to its capacity to store Carbon dioxide (CO2) from the atmosphere and fix nitrogen gas^2^. It may grow very fast under favourable conditions (moderate warmth and little sunlight) by using nitrogen for growth, carbon and sulphur for building proteins and phosphorous for DNA, RNA, and energy metabolism. utilization of biomass to produce high value products is attracting enhancement emphasis in In light of its fundamental importance in CO2 sequestration. Biomass valorization into various biobased products has been achieved in an integrated Azolla based biorefinery^25^.

To achieve a double benefit, agricultural waste can be modified into biocarbonaceous materials including activated carbon, graphene, and biochar via pyrolysis which participates Toward sustainable food waste management, and the created carbon-based materials can be employed as adsorbents for the elimination of bio-resistant pollutants^20–27^.

Biochar exhibits significant reactivity toward wastewater constituents and serves as an effective adsorbent in water purification owing to its high surface area and strong affinity for various functional groups^28^. Accordingly, it has attracted increasing attention for the removal of pollutants from wastewater. Its high adsorption capacity and natural availability make biochar a practical and environmentally friendly material for industrial wastewater treatment applications^29^.

Simultaneously, developing a high-capacity adsorption technique that is both economically feasible and environmentally friendly requires considerations that extend beyond merely identifying high-performance nano adsorbents. Further research is required to enable the adaption, reuse, and valorization of non-renewable adsorbents to promote circular economy principles. The reuse of wasted adsorbents has emerged as a prominent subject of exploration in the current research^30^. Many methods that facilitate the reapplication of aged adsorbents. Among these, reintegration into diverse applications such as catalysis and energy processing^31^.

The use of adsorbent materials as electrocatalysts for methanol oxidation has emerged as an interesting research area with the potential to produce cost effective and efficient electrode materials. Recently, Norhan et al. reported the use of (Fe/Si) oxide/P(oAP) for the adsorption of cobalt ions (Co2+). The resulting adsorbent (Fe/Si) oxide/P(oAP)/Co was subsequently reused as an electrocatalysts for methanol oxidation in direct methanol oxidation fuel cells (DMFCs). The (Fe/Si) oxide/P(oAP)/Co composite provided a greater number of active sites for electron transport, thereby enhancing the electrochemical current activity^32^.

The objective of this study is developing a highly efficient, high capacity, and cost-effective materials for the adsorption of cationic dye, with particular emphasis on MB removal. To achieving this objective, a bio adsorbent based on Azolla and biochar was synthesized through the combination of Azolla and biochar. This research focused on the following specific objectives (a) fabricate and characterize biomaterial Azolla/biochar (b) To investigate the adsorption kinetics, isotherms, and physicochemical properties related to MB removal by Azolla/biochar and (c) Reusing the adsorbent after MB adsorption as a node in Methanol oxidation fuel cell and focusing on theoretical predications, and practical validation.

## Materials and methods

### Materials

Azolla pinnata, ethanol, Ammonium persulphate (APS) (NH_4_)_2_S_4_O_8_ utilized as oxidizing agent and Poly vinyl alcohol (PVA) were obtained from LOBA CHEMEI. and isopropanol (98%) from fluka chemika. Hydrochloric acid (HCl), sodium hydroxide (NaOH, 99%), were purchased from biochemical in Egypt, and Biochar was obtained from private company.

### Extraction of azolla pinnata (AZ)

A field experiment was conducted to evaluate the beneficial effects of soil amendment with biochar in conjunction with inoculation of cyanobacteria (Tildeniella torsiva NA3 and Anabeana ferilissima) and the application of Azolla pinnata extract as a biofertilizer, in combination with the recommended mineral fertilizer on the performance of two wheat genotypes (Triticum aestivum cv. Sids 14 and sakha 95). The experimental field was prepared by plowing and puddling prior to cultivating. The experiment was arranged in a split plot design with three replicates. Each plot covered an area of 4.2 m2 (6 lines* 0.2 m row spacing * 3.5 m length), while the harvest area 2.8 m2 (4 lines* 0.2 m row spacing * 3.5 m length)^33^. all agronomic practices were performed in accordance with the protocols recommended by the Crop field research institute, agricultural research center.

### Incorporation of Biochar (BC)

The biochar was obtained from a private company, and its chemical composition was analyzed^34^, and presented in Table 1.

**Table 1:** Quantitative chemical composition and chemical constituents of prepared biochar including major and trace elements.

Type of analysis	Value
pH	8.6
EC dS/m	0.12
Total – nitrogen %	2.83
Potassium (K) %	0.15
Magnesium (Mg) %	0.16
Calcium (Ca) %	2.35
Silicon mg/kg	22.7
Organic matter %	3.7
Organic carbon %	2.51

### Fabrication of Azolla/biochar (AZ/BC) biomaterial

The (AZ/BC) biomaterial was synthesized using an ultrasonication technique. Initially, weight 0.5 g of extracted Azolla and 0.5 g of biochar were ground using a mortar and pestle. The resulting mixture was then dispersed in 50 mL of distilled water and subjected to ultrasonication for 4 h. subsequently the product was filtered and the obtained precipitate was dried at 50 º C for 24 h^35^.

### Preparation of the working electrode

After the (AZ/BC) biomaterial was applied for MB dye adsorption, the residual material was used for the preparation of the working electrode in DMFCs application. Initially the residue was mixed with PVA and ground using a mortar and pestle. Subsequently the mixture was dispersed in a 1:2 (v/v) isopropanol/distilled water solution and sonicated for 1 h at ambient temperature to obtain a homogeneous suspension. Thereafter the uniform suspension was deposited onto graphite sheet using a micropipette. The modified graphite sheet was dried at 50º C for 24 h until completely dry prior to its use in electrochemical measurements^36^.

### Characterization of Azolla, (AZ/BC) biomaterial and (AZ/BC) biomaterial/MB

The crystal structure, purity of the synthesized materials, and phase composition were examined by X-Ray Diffraction (XRD) using a PANalytical Empyreen diffractometer (Netherlands) equipped with Cu-Ka radiation operated at 40 kV and 35 mA, with a wavelength of 1.54045 Å. Fourier transform infrared (FTIR) spectra were recorded using a Vertex 70 spectrometer (Burker, Germany) employing the KBr pellet method over the wavenumber range of 400–4000 cm-1 to identify the functional groups present in the materials. The pH of the solution was measured using 1030 pH meter. The microstructural features and surface morphology of the samples were investigated by scanning electron microscopy (SEM) using s ZEISS Sigma 500 VP microscope.

### Determination of optimal MB adsorption conditions

The experiment was conducted and analyzed the removal of MB dye at ambient temperature. Adsorption experiments of MB dye onto the proposed biocatalysts were carried out using a batch method. A stock solution was prepared by dissolving 0.1 g of MB dye in 1000 ml of dist. Water to obtain a concentration of 100 ppm under continuous shaking at 400 rpm. This stock solution was then diluted to 10 ppm in a 50 ml MB solution to determine the optimum adsorption conditions, including effect of contact time, initial concentration of MB dye, solution pH, and adsorbent dosage.

The effect of contact time on adsorption performance was examined over an interval of 10–360 min. the influence of AZ/BC biomaterial dosage was assessed in the range of 0.025–0.125 g using a 50 ml of a 10 ppm MB dye solution. The pH solution was determined by adding 0.1 N HCl or 0.1 N NaOH with pH range of 3.42 to 9.17. Additionally, the influence of the initial MB dye solution concentrations on adsorption performance was also analyzed using concentrations from 5 to 70 ppm and the effect of temperature range from 298 to 333 K. The adsorption removal efficiency (R%) and adsorption capacity (qe, mg/g) were determined according to Eqs. (1) and (2) respectively. All adsorption measurements were conducted three times independently and the average results are provided with statistical error bars with 3 reproducibility.

1 $$\:Removal\:Precentage\left(\mathrm{\%}R\right)=\frac{{A}_{i}-{A}_{f}}{{A}_{i}}\mathrm{*}100$$

2 $$\:{q}_{e}=\frac{{A}_{i}-{A}_{f}}{m}\mathrm{*}V$$

### Electrochemical measurements

Electrochemical measurements were performed using an OrigaFlex potentiostat (OrigaLys ElectroChem OGF01A, Rillieux, France). All experiments were performed at room temperature using two electrode configurations comprising a working electrode and a counter electrode. A platinum (pt) sheet served as the counter electrode while the modified (AZ/BC)/MB biomaterial electrode was used as the working electrode. A solution of 0.5 M Na_2_S_2_O_8_ was employed as electrolyte under conditions without and with methanol to analyze the electrocatalytic performance of the fabricated electrode. Chronoamperometry (CA) measurement was conducted at applied 1 V for 3600 s while the cyclic voltammetry (CV) measurements were measured at scan rates from 10 to 100 mV/s. An electrochemical impedance spectroscopy (EIS) investigation was done A 1 V with 10mV amplitude at fixed frequency.

### Computational study for (AZ/BC) biomaterial

The study examined the adsorption of MB Dye on the (AZ/BC) biomaterial surface and the associated desorption sites by Monte Carlo (MC) simulation. The Monte Carlo simulation utilized the adsorption Locator module, employing the universal force field with current charges specified in the charges section. This work was guided by the fundamental concepts of Monte Carlo simulation, as delineated by Frenkel and Smit. In the MC simulations, the electrostatic and van der Waals interactions were managed using Ewald and group-based methodologies, respectively.

## Results and discussion

### Characterization of the obtained materials

#### The surface morphology study

**Fig. 1:**
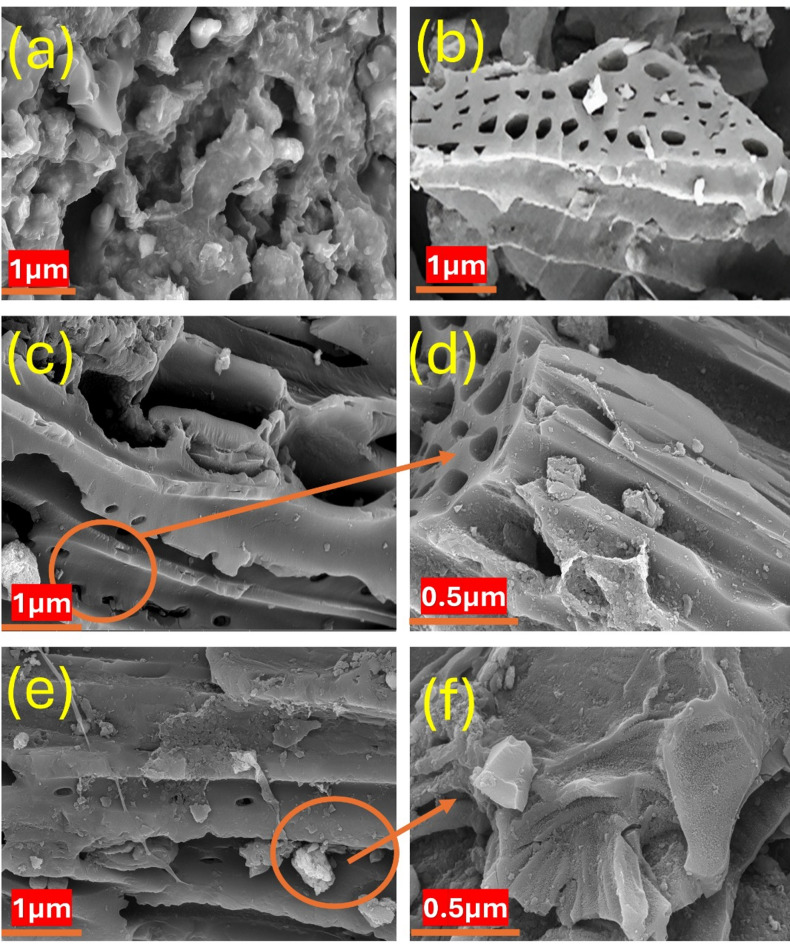
SEM micrograph illustrating the surface morphology (**a**) Azolla extract (AZ) (**b**) biochar (BC), (**c**,** d**) (AZ/BC) biomaterial composite at different magnification, and (**e**,** f**) (AZ/BC) biomaterial/MB at different magnification.

Scanning electron microscopy (SEM) observation ware conducted to elucidate the structural morphology of the Azolla extract, Biochar, (AZ/BC) biomaterial and (AZ/BC) biomaterial/MB). Figure 1(a) illustrates an SEM topographic image of Azolla extract which shows a rough surface with many variable pores with different size which distributed on the surface. The average size of pores was between 500 nm and 600 nm. While the surface structure of biochar appeared as highly porous surface which render the surface highly qualified to adsorb many pollutants as shown in Fig. 1(b). However, formation of the biomaterial with biochar led to noticeable alterations in its structural morphology. the stacked layers appeared with uniform pores size ranged 150–200 nm as shown in Fig. 1(c, d). Moreover, After MB dye adsorption, the cavities of pores and groves in (AZ/BC biomaterial was filled with MB dye which was shown in (Fig. 1e, f). Notably, noticeable aggregations in the (AZ/BC) biomaterial/MB) as well as enhanced particle size, confirm the successful adsorption of MB dye on the surface of (AZ/BC) biomaterial^37^.

#### The XRD pattern study

**Fig. 2:**
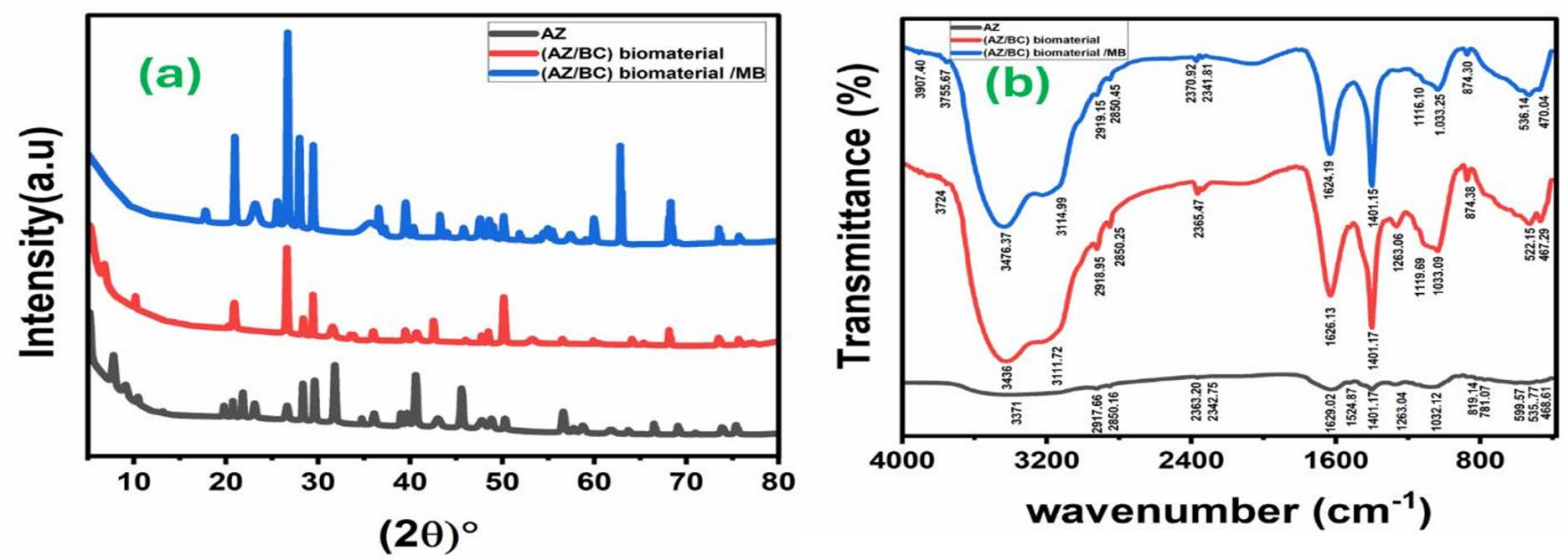
(**a**) XRD pattern, and (**b**) FTIR spectra, including Azolla extract (AZ), (AZ/BC) biomaterial and (AZ/BC) biomaterial/MB.

X-Ray diffraction (XRD) studies indicate that modification in the crystalline structure of the Azolla pinnate adsorbent. XRD analysis shows that well defined peaks are associated with crystalline phase, while broad peaks indicate the amorphous character of the plant. The XRD pattern of raw Azolla biomass Fig. 2a demonstrated the existence of strong peaks at 2θ 8^o^, 24 ^o^ 29 ^o^, 31 ^o^, 44 ^o^, 46 ^o^, 56 ^o^, and 75^o^ that can be related to the presence of complex minerals in the structure of the biomass. While XRD of biomaterials containing BC and AZ extract, there are noticeable sharp peak at 25 ^o^ and44 ^o^ which was characteristics to biochar and some peaks of azolla shifted and the intensity decrease which indicated to formation of biomaterial^37,38^. While, after MB dye adsorption, the diffraction peaks Upon adsorption of MB onto (AZ/BC) biomaterial, the diffraction peak at 2θ = 23.8° shifted to the right, and the peak width increased, supposing the crystallinity was modified after adsorption of MB by (AZ/BC) biomaterial, which showed that MB was successfully adsorbed by (AZ/BC) biomaterial^39^.

#### The FTIR study

The highest wavenumber recorded in the hot water extract of Azolla nilotica exhibited peaks at 3655 cm^−1^signifying the presence of robust O-H bonding in carboxyl groups^40^. The peak appears at 939 cm^− 1^ indicates to the bending of O-H groups. While the peak appears to 2920 cm^− 1^ signifies the -CH stretching of aliphatic groups^41^. The bonds of COO anions and conjugated C = C aromatic structures appear at 1619 cm^− 1^. The C = O groups from conjugated aromatic rings indicated at1 462 cm^− 1^. Additionally, the bending vibrations of the C-OH alcoholic group and C-O single bond vibrations of ether linkages appear at 1400 cm^− 1^ reflects^42^. Moreover, according to prior literature, functional groups such as C-O, OH, C = O, and COOH exhibit a great propensity to bind metals resulting in the formation of very stable nanoparticles^43^. IR band signifies several phytochemicals present in the Azolla nilotica extract including those inadequately assessed by reagent-based phytochemical screening methods.

Figure (2b) shows the FTIR spectra of PSD, SSD, and SDO biochar after adsorbed with MB dye for 3 h. It was revealed in all the FTIR analyses of all tested biochar after they had been treated to an MB dye removal method that there were bands at 1591, 1485, 1392, and 1340 cm^− 1^, which match to the MB dye.

### Adsorption of MB on biomaterials

#### Influence of contact time

**Fig. 3:**
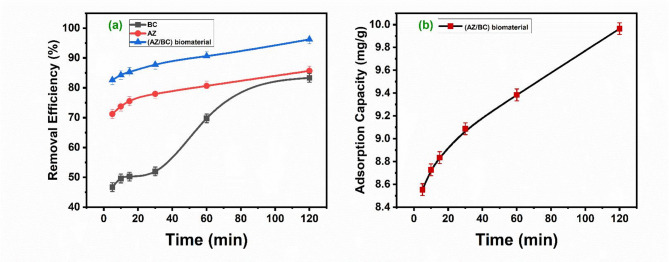
(**a**) Influence of contact time on the adsorption of MB dye for BC, AZ, and AZ/BC biomaterial composite expressed as removal efficiency (%), and (**b**) time dependent adsorption capacity (mg/g).

The influence of contact time on the adsorption MB was evaluated using a 0.025 g adsorbent dosage in 25 mL MB as 5ppm concentration for AZ, BC, or (AZ/BC) biomaterial beads across various time intervals ranging from 5 to 120 min. Figure (3a) indicates that the initial rate of MB adsorption escalates with increase contact time. Initially, the MB dye adsorption rate remains constant which was attributable to the significant interaction occurring between the adsorbate and the unoccupied adsorptive sites on the adsorbent surface and then saturation^44^. In the case of AZ, BC, and (AZ/BC) biomaterial, the lowest removal of adsorbent was 46.71%, 71.22%, and 82.58% at 5 min, respectively, and it reached a maximum (83.31%, 85.67%, and 96.21%) after 120 min, respectively. The adsorption capacity of the (AZ/BC) biomaterial increases from 8.55311 mg/g to 9.96431 mg/g when the contact time increases from 5 min to 120 min is shown in Fig. (3b). Therefore, the most effective adsorption contact time for of MB dye is 120 min for (AZ/BC) biomaterial.

#### Kinetics study for MB adsorption

Adsorption kinetics illustrates the relation between contact time and adsorption capacity are related according to mass transfer, diffusion rates, dynamic equilibrium, and adsorption rate^45,45^. Examination of these factors provides a clearer understanding of the adsorption process and its underlying mechanisms. Consequently, the experimental adsorption data were analyzed using the Pseudo first order, pseudo second order, the intraparticle diffusion model to describe adsorption rate^46^, and the Elovich model to describe the adsorption rate which decline exponentially with increasing in the adsorbed particle were employed to fit the experimental adsorption results^47^.

**Fig. 4:**
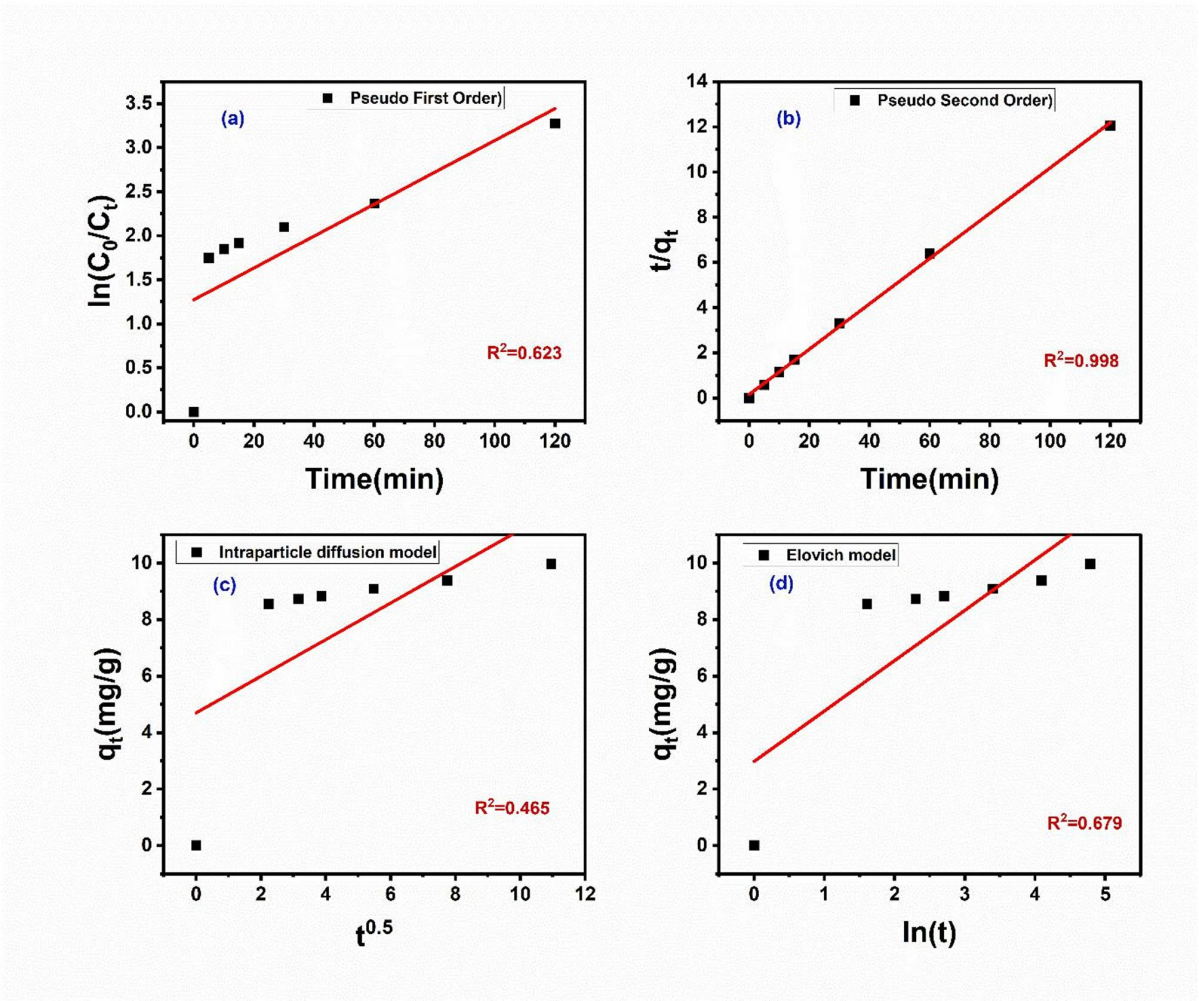
Kinetic modelling of MB dye adsorption onto (AZ/BC) biomaterial composite using (**a**) Pseudo first order, (**b**) Pseudo second order, (**c**) intraparticle diffusion, and (**d**) Elovich kinetic models.

The pseudo-first-order model’s linear form is displayed in Eq. (3) below^48^.

3 $$\:\mathrm{ln}\left(\frac{{C}_{0}}{{C}_{t}}\right)=\:{k}_{1}t\mathrm{*}C$$

where C_0_ is the pseudo-first order rate constant, C_t_ k_1_ (min^− 1^), and t is the duration in minutes. Plotting ln (C_0_/C_t_) linearly across time is one method of analysing the rate constant k_1_^48^.

The following Eq. (4) provides the linear expression for the pseudo-second-order kinetic model^49^:

4 $$\:\frac{t}{{q}_{t}}=\:\frac{1}{{K}_{2}{q}_{e}^{2}}+\frac{1}{{q}_{e}}t$$

where q_e_ (mg/g) is the quantity of MB degradation at equilibrium, q_t_ is the solute’s adsorption capacity on the adsorbent at time t (mg/g), and k_2_ is the equilibrium rate constant of pseudo-second order adsorption (mg/g/min)^49^. To determine the values of q_e_ and k_2_, the slope and intercept of the linear plot of t/q_t_ against t were utilized.

The experimental data were further evaluated using the intraparticle diffusion model to explain the diffusion mechanism of MB dye on the surface of (AZ/BC) biomaterial. This model is expressed by Eq. (5)^50^.

5 $$\:{q}_{t}=K\mathrm{*}{t}^{0.5}+C$$

where C is the boundary layer thickness and K is the intraparticle diffusion constant (mg.min^− 1/2^.g^− 1^). The solitary regulating step at C = 0 is intraparticle diffusion. Adsorption consequently takes place inside the adsorbent^50^.

The Elovich equation is a kinetic model that describes a chemisorption mechanism, in which the adsorption rate exhibited an exponential decline with increasing the adsorbed. This model is commonly represented by Eq. (6)^47^.

6 $$\:{q}_{t}=\left(\frac{1}{\beta\:}\right)\:\mathrm{*}\:\mathrm{ln}\left(\alpha\:\beta\:\right)\:+\:\left(\frac{1}{\beta\:}\right)\:+\mathrm{l}\mathrm{n}\left(t\right)$$

where (*α)* is defined as the initial adsorption rate (mg/g.min), whereas β corresponds to the desorption constant (g/mg). The inverse of *β* reflects the availability of adsorption sites, and the adsorbed quantity at zero *t* is expressed as $\:\frac{1}{\beta\:}\mathrm{l}\mathrm{n}\left(\alpha\:\beta\:\right)$^47^.

The linear graphs for all studied kinetic models are depicted in Fig. 4(a-d). the associated parameters including the (R^2^) correlation coefficients values were calculated for each model and are provided in Table 2. The R^2^ values corresponding to the pseudo first order, pseudo second order, interparticle diffusion, and Elovich models were 0.623, 0.998, 0.465, and 0.679 respectively. The pseudo second order model exhibited the highest correlation coefficient for MB adsorption compared to the other models, indicating a superior fit plot compared to the other models. the maximum adsorption capacities calculated from the pseudo second order closely matched the experimental values. These studies indicating that the adsorptions of MB dye and crystal follow a pseudo-second order mechanism.

**Table 2:** parameters of kinetic modelling of MB dye adsorption onto (AZ/BC) biomaterial composite using (a) Pseudo first order, Pseudo second order, intraparticle diffusion, and Elovich model.

Kinetics models	Parameters	Values
The pseudo-First-order	$\:k$	0.01808
	R^2^	0.62371
The pseudo-second order	q_e_(mg/g)	9.976057462
	q_e_^2^(mg/g)	99.521722
	K_2_	0.063486811
	R^2^	0.99891
Intraparticle diffusion model	$\:\mathrm{K}$	4.70011
	C	0.64726
	R^2^	0.46525
Elovich model	$\:1/{\upbeta\:}$	1.78265
	$\:(1/{\upbeta\:})\:\mathrm{*}\:\mathrm{l}\mathrm{n}\left({\upalpha\:}{\upbeta\:}\right)$ R^2^	2.97903
		0.67929

#### Effect of concentration of MB dye of (AZ/BC) biomaterial composite

**Fig. 5:**
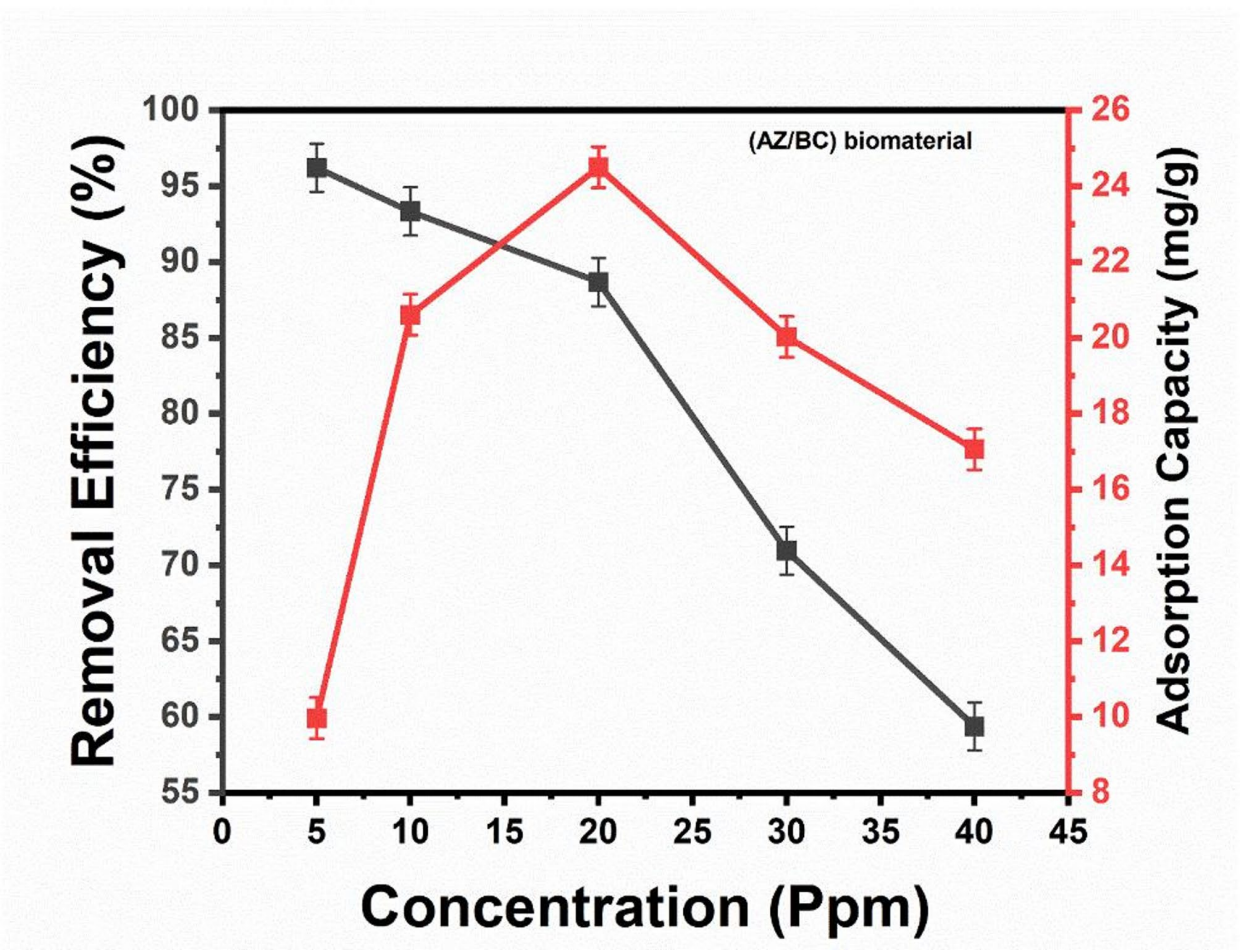
Effect of initial MB concentration on the adsorption performance of (AZ/BC) biomaterial composite, showing both removal efficiency (%) and adsorption capacity (mg/g).

**Fig. 6:**
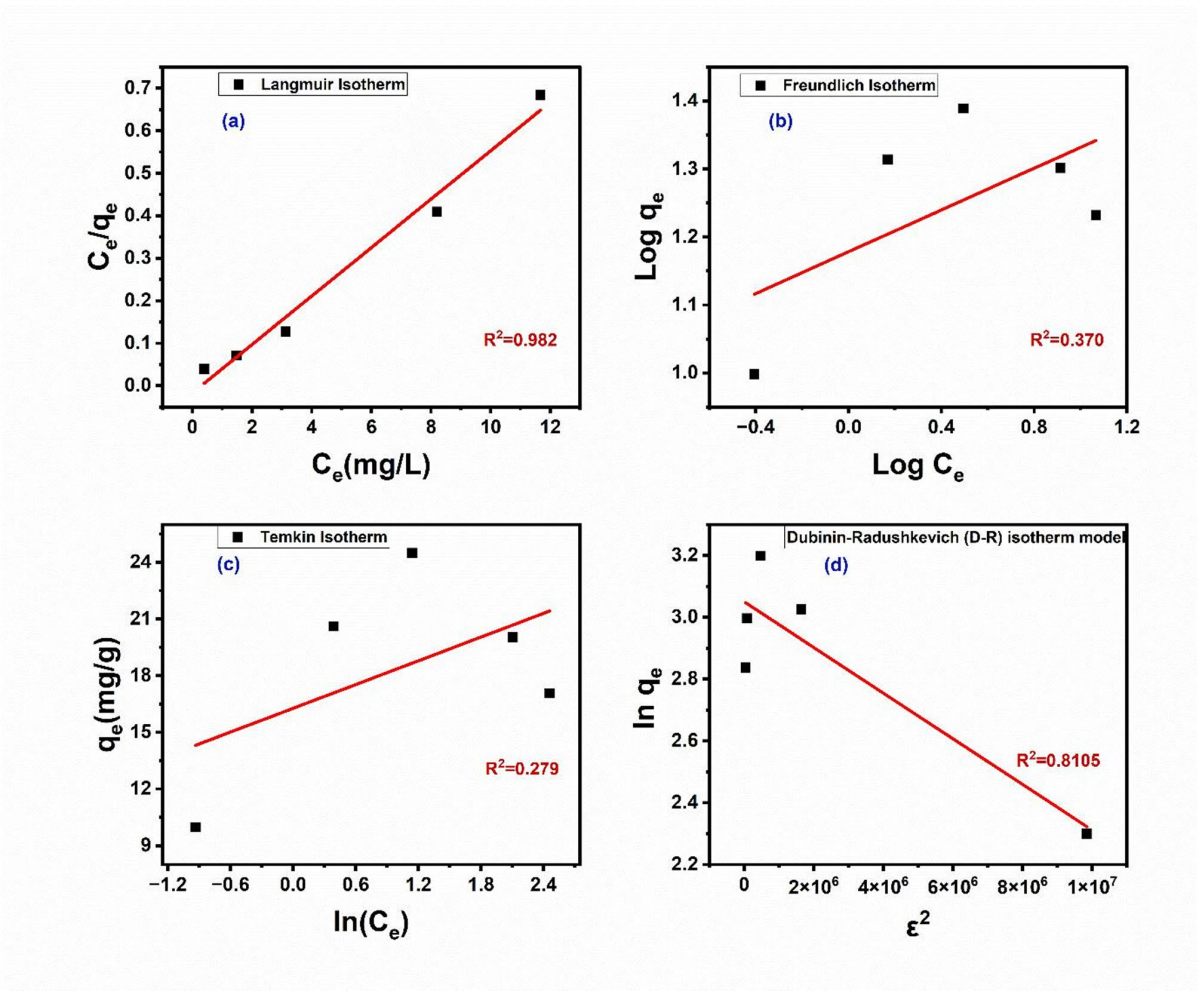
Isothermal modelling of MB dye adsorption onto (AZ/BC) biomaterial composite using (**a**) Langmuir, (**b**) Freundlich, (**c**) Temkin, and (**d**) Dubinin-Radushkevich (D-R) isothermal models for MB adsorption on AZ/BC biomaterial.

Five various beginning concentrations (5, 10, 20, 30, and 40 ppm) of MB were chosen to assess the effect of the initial MB dye concentration on the adsorption capacity of the (AZ/BC) biomaterial and removal efficiency is shown in Figs. 5, 6. All experimental adsorption conditions were kept constant including the use of 25 ml of 5 ppm MB solution and 0.025 g of (AZ/BC) biomaterial at room temperature. Figure 5 explains the analyses percentage of dye removal (% Removal) in relation to the initial MB dye concentration (C_o_) and adsorption capacity (q_e_). The proportion of removal of MB dye has been demonstrated to decline from 96.21% to 59.37% for MB when the original dye concentration increases from 5 to 40 ppm. Additionally, the result may reflect a higher availability of surface active sites at reduced dye concentrations^51^. Hence, the adsorption removal increases due to the interaction between MB dye molecules and the biomaterial (AZ/BC)^52^. The observed decline in adsorption at increased dye concentrations is attributed to fewer active sites and illustrates the growth of q_e_ (mg.g^− 1^) from 9.964 to 17.057 mg/g with the raising of the initial concentration of MB dye from 5 to 40 ppm respectively. This behaviour can be attributed to the increased adsorption rate and gradual occupation of all available adsorption sites at higher concentration, leading the system toward equilibrium^53^. Therefor the optimal working concentration for MB dye adsorption was determined to be 5 ppm.

#### Isotherms study for MB adsorption

The adsorption isotherm models are fundamental for describing the interaction between adsorbate and adsorbent. The Langmuir (Eq. (7), Freundlich (Eq. (8)) and Tamkin (Eq. (9)) isotherms are among the most widely applied models for explain adsorption isotherms.

**Table 3:** Parameters of isothermal modelling of MB dye adsorption onto (AZ/BC) biomaterial composite using Langmuir, Freundlich, Temkin, and Dubinin-Radushkevich (D-R) isothermal models for MB adsorption constants on AZ/BC biomaterial.

Isothermal models	Parameters	Values
Langmuir Model	q_m_(mg/g)	20.588
	b	26.83425
	R^2^	0.983
Freundlich Model	1/n	0.15349
	n	6.52
	K_f_ (L/mg)	15.076134
	R^2^	0.37043
Temkin model	β	2.09577
	ln(A_T_) (J/mol)	7.762936773
	R^2^	0.27914
Dubinin-Radushkevich (D-R)	q_max_(mg/g)	21.11218
	β	7.39 × 10 − 8
	E (kJ/mol)	2.6015709
	R^2^	0.81058

Based on the Langmuir isotherm model, adsorption occurs as a monolayer of sorbate molecules on the surface of the sorbent which possesses a fixed number of equivalent sites that are similarly energetic. Equation (7) represents the Langmuir isothermal^54^:

7 $$\:\frac{{C}_{e}}{{q}_{e}}=\:\frac{{C}_{e}}{{q}_{m}}\mathrm{*}\:\frac{1}{{b\mathrm{*}q}_{m}}\:$$

where b is the Langmuir constant (L/mg), which represents the free energy of sorption and the affinity of accessible sites. The dye concentration on the sorbent at equilibrium is represented by q_e_ (mg/g). Ce is the dye’s equilibrium concentration in solution (mg/L). When a monolayer is applied to the sorbent, the dye concentration is represented by qm (mg/g)^54^.

Equation (8) provides the Freundlich equation for heterogeneous surface energy systems^55^.

8 $$\:\mathrm{log}{q}_{e}=\mathrm{log}{k}_{f}+\frac{1}{n}\mathrm{log}{C}_{e}$$

where the plot of log q_e_ versus log C_e_ yields the Freundlich constants K_F_ and n. The system’s sorption capacity and intensity are linked to the parameters K_F_ and 1/n. The availability of the sorbent/adsorbate systems is represented by the magnitude of the term (1/n)^55^.

The linear Temkin equation form is stated by the following equation (Eq. (9)^46^

9 $$\:{\mathrm{q}}_{e}={\upbeta\:}{\mathrm{l}\mathrm{n}(A}_{T})+\beta\:\mathrm{ln}({C)}_{e}$$

Where Eq. (10)

10 $$\:\beta\:=\frac{RT}{b}$$

R corresponds to the universal gas constant (8.314 J/mol K), b represents the Temkin constant associated with the heat of sorption (J/mg), and T refers to the absolute temperature in Kelvin. The slope and intercept of q_e_ plotted against lnC_e_ are used to calculated the Temkin constants (A_T_) and b^46^.

The Dubinin-Radushkevich (D-R) isotherm model represented an empirical adsorption model typically utilized to elucidate adsorption mechanisms characterized by a Gaussian distribution of adsorption energy on heterogeneous surfaces. This isotherm is primary applicable to intermediate adsorbents concentration owing to its unrealistic asymptotic behaviour. The model is a semi-empirical adsorption expression that incorporates a pore-filling mechanism combined with adsorption. It assumes a multilayer structure characterized by Van Der Waals forces relevant to physical adsorption processes and serves as a fundamental Eq. (11) that qualitatively explains the adsorption phenomena of gases and vapours by microporous adsorbent. This linear D-R model is articulated as^56,57^

11 $$\:\mathrm{ln\:}\mathrm{q}\mathrm{e}\mathrm{\:=\:ln\:}\mathrm{q}\mathrm{max}\mathrm{\:-\:}{\beta\epsilon}\mathrm{2}$$

The adsorption capacity is specified by the parameter q_max_ (mg/g), the adsorption energy is connected to β (mol^2^/kJ^2^), and the Polanyi potential for equilibrium concentration is ε (kJ/mol), which reflects the transfer of 1 mol of MB dye into the adsorbent surface. The following expression represents this Polanyi potential Eq. (12)^57^:

12 $$\:{\epsilon\:=\:}\mathrm{RTln}\mathrm{[\:1\:+\:(\:}\frac{1\text{}}{\mathrm{C}\mathrm{e}}\mathrm{)]}\text{}$$

A constant E (kJ/mol) is added to the D-R isotherm process to characterize the type of adsorption or the nature of adsorbate-adsorbent bonding. This is known as the adsorption free energy, and it is the change in free energy that occurs when one mol of ion is transported into the adsorbent surface. In terms of the adsorption energy constant, this adsorption free energy can be represented as Eq. (13)^58^

13 $$\:E=\:\frac{1}{\sqrt{2\beta\:}}$$

Through the parameter (E) it is possible to determine the types of adsorption interaction forces that interaction between adsorbate molecules and adsorption sites. This parameter calculated whether the adsorbate molecules adhere to the adsorbent surface through an ionic exchange mechanism (chemical process), or a physical interaction mechanism. An ionic exchange mechanism must facilitate the adsorption if the adsorption energy values between 8 and 16 KJ/mol. Physical adsorption occurs when (E) is less than 8 kJ/mol, whereas chemical adsorption takes place when (E) exceeds 16 kJ/mol. The linear relationship of ln q_e_ versus ε_2_ for MB adsorption on the (AZ/BC) biomaterial surface is illustrated in Fig. 6d. The value of β (mol²/kJ²) is derived from the slope of the straight line, while q_max_ is calculated from the intercept. Table 3 displays the values obtained by the D-R isotherm method. The value of β has been employed to ascertain the E value, which is calculated to be 2.6015709 kJ/mol. The physical-sorption process is the mechanism via which adsorption occurs^59^.

Figure 6(a-d) and Table 3 are presented the equilibrium parameters obtained from the Langmuir, Freundlich, Temkin, and Dubinin-Radushkevich (D-R) isothermal models for MB dye adsorption onto the (AZ/BC) biomaterial surface. The various constant and parameters for each isotherm were determined using linear regression plots. All obtained values indicate the farvoable adsorption of MB dye onto the (AZ/BC) biomaterial. The calculated isotherm parameters are summarized in Table 3. For the Langmuir model, the correlation coefficient (R2) was higher than those of the Freundlich, Temkin, and D-R models with values of 0.983, 0.370, 0.279, and 0.810 respectively. The Langmuir model is therefore the most effective technique to describe how MB adsorbs onto the (AZ/BC) biomaterial and given that the greatest adsorption amount (q_m_)for MB dye was 20.588 mg/g, this meaning that the adsorption occurs as a monolayer of sorbate molecules on the surface of the sorbent which possesses a fixed number of equivalent sites that are similarly energetic. Additionally, the Freundlich 1/n value of 0.153 is less than 1 further confirmation the best adsorption conditions. Overall, the Langmuir isothermal exemplifies an efficient adsorption process for MB onto the (AZ/BC) biomaterial surface.

#### Effect of pH and adsorbent dosages

**Fig. 7:**
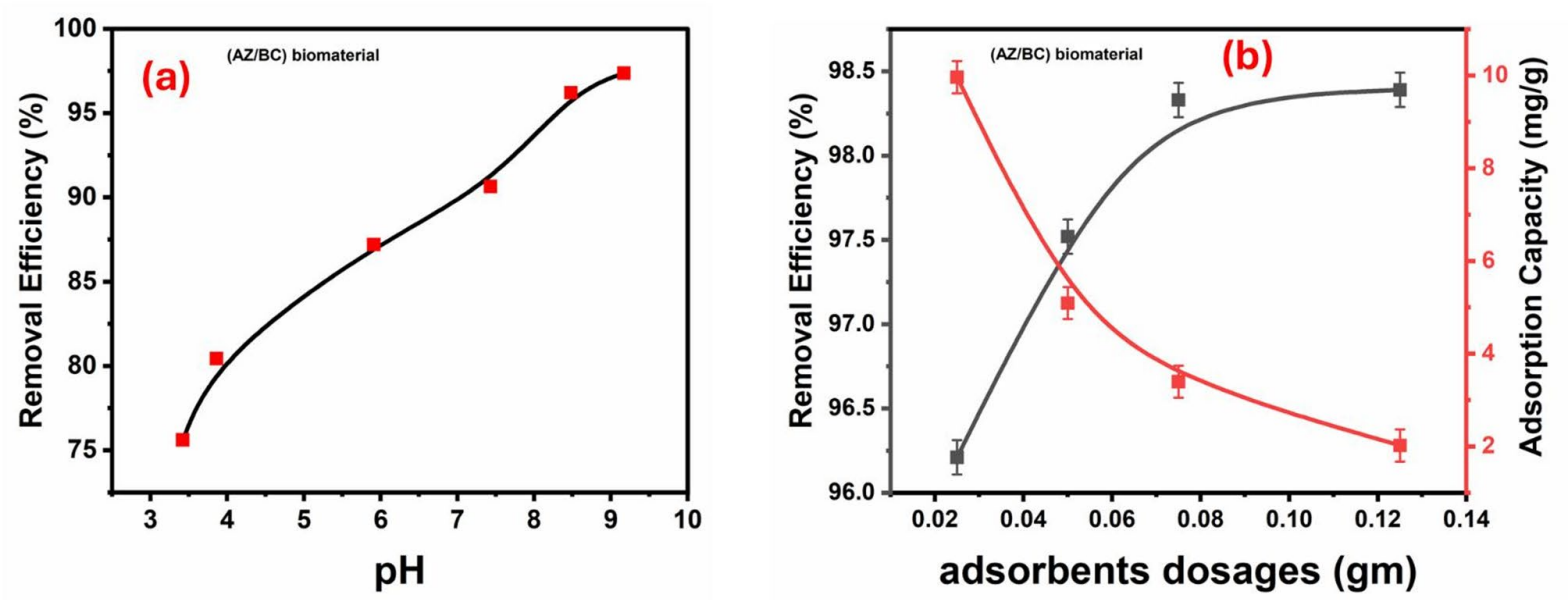
Effect of (**a**) initial solution pH, and (**b**) AZ/BC biomaterial dosage on the adsorption performance of MB expressed in terms of removal efficiency and adsorption capacity (mg/g).

pH of solution strongly impacts the adsorption and MB removal. This effect allows to ascertain the cationic behaviour in the pH range (0–6.9) and anionic properties range (7.1–14) of the adsorbent and adsorbate. The impact of solution pH on the adsorption of MB dye onto (AZ/BC) biomaterial surface was examined within the pH range of 3.42 to 9.17, under the experimental conditions of with an initial MB concentration of 5 ppm at 25 mL volume, and (AZ/BC) biomaterial dosage of 0.025 g. As demonstrated in Fig. 7a, the adsorption percentage of the (AZ/BC) biomaterial for MB dye increased from 75.61% to 97.37%, from 3.42 to 9.17 as pH increased respectively. A decrease in MB adsorption at low pH (Acidic) is attributed to the competition between cationic MB molecules and excess H^+^ ions in the solution for active sites on the (AZ/BC) biomaterial^60^. Additionally, the adsorption rate is decreased by the MB molecules repulsion with H^+^ ions. Increasing the pH solution (Alkaline) enhanced the number of negatively charged sites, which are suited for adsorption^53^. In the current experiment, maximal MB adsorption was detected at pH 9.17, and consequently pH 9.17 was selected for continued adsorption study.

#### Effect of dosage from biomaterials

The adsorbent dosage is a notable component of the adsorption process. The initial (AZ/BC) biomaterial amount, ranging from 0.025 to 0.125 g in 25 ml of a 5 ppm MB concentration at room temperature was used to evaluate the effect of dose on the dye elimination percentage, as demonstrated in Fig. 7b. Results indicated that the removal percentage increased from 96.21% to 98.39% with the adsorbent dosage of the (AZ/BC) biomaterial increased from 0.025 to 0.125 gm, respectively, while, the adsorption capacity of MB declined from 9.96431 to 2.01992 mg/g. This is linked to the higher dosage that raises surface area, as well as the availability of numerous active sites for MB molecule adsorption^61^. Therefore, the optimum dose of (AZ/BC) biomaterial for eliminating MB dye was selected to be 0.025 g in a 25 ml volume for subsequent studies.

#### Effect of Temperature and thermodynamic study for AZ/BC biomaterial

**Fig. 8:**
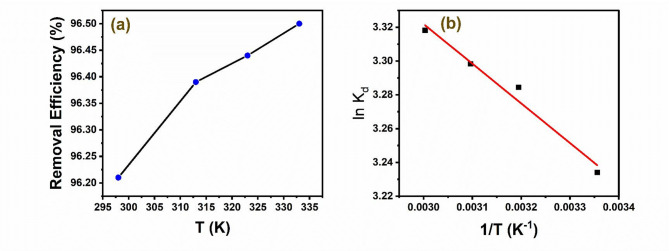
(**a**) Effect of temperature, and (**b**) Van’t Hoff plot for the adsorption of MB dye onto the surface of AZ/BC biomaterials.

In Fig. 8a illustrates the effect of temperature on MB dye adsorption. As shown in Fig. 8a, increasing the temperature from 298 K to 333 K resulted in a higher adsorption efficiency, this indicating that the adsorption of MB is an endothermic process.

The thermodynamic parameters values in adsorption systems represent the reliable indicators for evaluating the practical applicability of the adsorption process^62^. The adsorption of MB dye onto AZ/BC biomaterial adsorbents was investigated over a temperature range of 283–333 K. are shown in Fig. 8b. The Gibbs free energy change (∆Gº) was calculated using Eq. (14)^63^:

14 $$\:{\Delta\:}{G}^{0}=-RTln{K}_{L}$$

Where R, T, and Kd denote the gas constant (8.314 J. mol^− 1^.K^− 1^), the absolute temperature (K), and the equilibrium adsorption constant respectively^63^. Equation (15) were applied to determine the enthalpy (∆Hº) and entropy (∆Sº) values (Kj/mol)^64^. the thermodynamic parameters ∆Hº and ∆Sº were calculated from the slope and intercept of the vant’t Hoff linear plot of ln Kd versus 1/T^65^ and are illustrated at Table 4.

15 $$\:ln{K}_{\mathrm{d}}=\frac{{{\Delta\:}\mathrm{S}}^{0}}{R}-\frac{{{\Delta\:}\mathrm{H}}^{0}}{RT}$$

The results of the thermodynamic modelling at different temperatures are presented in Table 4. The negative values of ∆Gº indicate that the process is feasible and spontaneous. The ∆Gº values decreased from − 6.79 to −9.18 KJ/mol with increasing temperature demonstrating that MB removal using the AZ/BC biomaterial becomes more favorable. The calculated ∆Hº and ∆Sº values were 1.95 and 0.003 KJ/mol respectively. the positive ∆Hº value confirms that the adsorption process is endothermic^61^. Moreover, the positive ∆Sº value suggests structural changes in the adsorbent during MB dye uptake and reflects an increased degree of randomness at the solid liquid interface^66^. This positive value also indicates a strong affinity of the AZ/BC biomaterial toward MB^61,67^.

**Table 4:** parameters for MB adsorption on AZ/BC biomaterial, standard enthalpy, entropy, and free energy changes were calculated.

Temperature (K)	ΔG^0^ (J/mol)	ΔH^0^ (J/mol)	ΔS ^0^ (J/mol K)
283 to 333	−8012 to −9186	1953.009	33.478

#### Regeneration performance of AZ/BC biomaterial

**Fig. 9:**
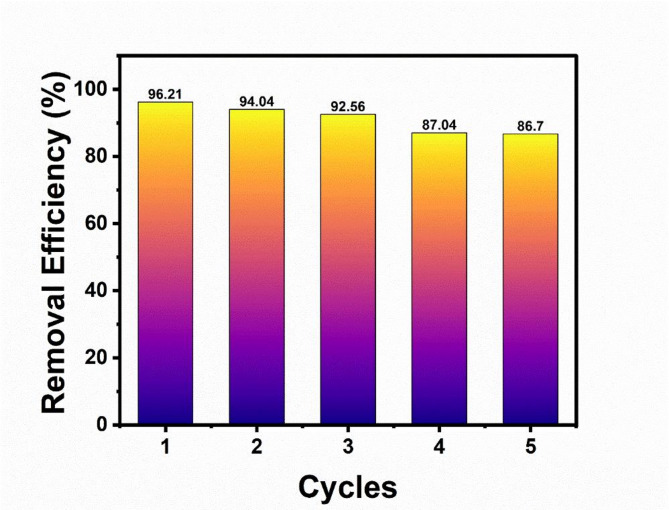
Regeneration studies of MB dye adsorption over AZ/BC biomaterial. Temperature: 25 °C; adsorbent-to-solution ratio: 0.025 g/25 mL; concentration of MB: 5 ppm; contact time: 120 min.

Regeneration experiments were performed to evaluate the practical applicability of the AZ/BC biomaterial using an MB dye solution concentration of 5 ppm. The AZ/BC biomaterial was tested through five successive adsorption regeneration cycles, and the obtained results are presented in Fig. 9. A slight reduction in the maximum adsorption efficiency of MB onto the AZ/BC biomaterial was observed with an increasing number of adsorption and regeneration cycles. This decline may be attributed to a decrease in the surface area of the AZ/BC biomaterial and weight loss occurring during the recovery pf the AZ/BC biomaterial throughout the adsorption regeneration process. As shown in Fig. 9 the fresh AZ/BC biomaterial exhibited an adsorption efficiency of 96.21% for MB dye, which decreased to 86.7% after the 5th cycle. The material demonstrated good recyclability, suggesting that it can be effectively reused for multiple cycles in dye removal applications for wastewater treatment.

### Valorization of (AZ/BC) biomaterial/MB for electrochemical activity in Methanol oxidation fuel cell

#### Cyclic voltammetry (CV)

**Fig. 10:**
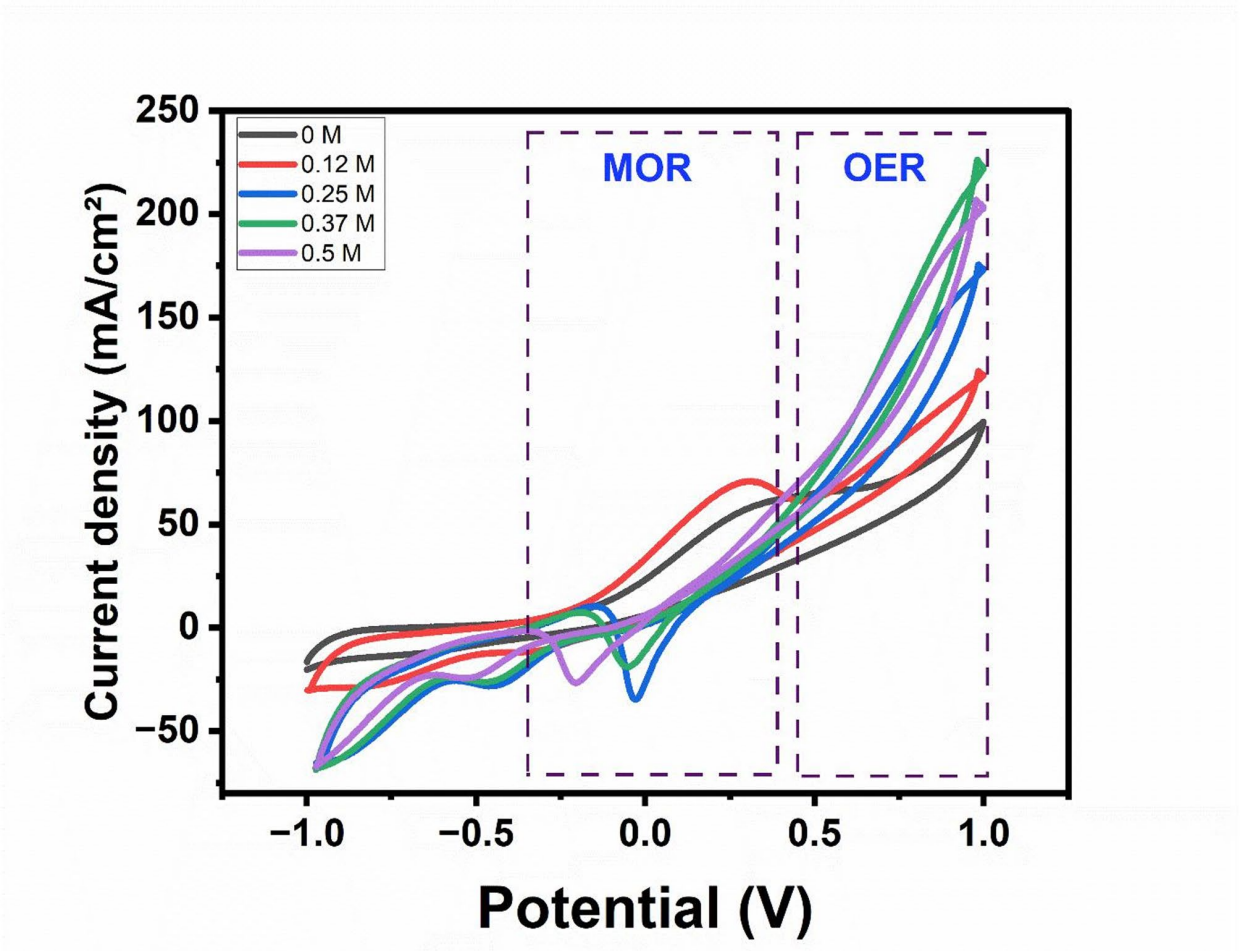
CV curves of AZ/BC biomaterial/MB electrode at different methanol concentration, recorded to evaluate the electrochemical behaviour of the modified electrode.

In Fig. 10 demonstrates electrochemical (AZ/BC)/MB behaviour in a 0.5 M Na₂S₂O₈ solution with and without methanol. At absence of methanol, CV graph exhibited a low current density with one anodic peak with no characteristic redox peaks, which indicated the baseline electrochemical response of the catalyst in the Na₂S₂O₈ solution. The response was dominated by capacitive currents and a very small degree of redox activity on the surface of the catalyst which might have been influenced by the oxidative nature of Na₂S₂O₈. The behaviour of the (AZ/BC)/MB and its reaction rate at the electrode solution interface examined how varied concentrations of methanol alter. There are considerable rise in the anodic oxidation peak current density from99.738 mA/cm² to 226.55 mA/cm² when methanol concentration increased from 0 M to 0.37 M. This jump is apparently because there are more methanol molecules on the catalyst surface which speeding up the process of electro-oxidation in the presence of Na₂S₂O₈ solution and perhaps in the production of active catalytic species^68,69^. Notably, the anodic peak current density reached to 203.21 mA/cm² at a methanol concentration of 0.5 M, as observed in Fig. 10. The plateau illustrates that methanol and its intermediates including CO or other carbon-containing species overload the catalytic surface and consequently, methanol attachment and oxidation are restricted further^70,71^.

When methanol was added, there were clear anodic and cathodic peaks, which showed that methanol was being oxidized electrocatalytically. The anodic peak shows direct oxidation of methanol on the catalyst surface, and this is seen in the potential range of 0.12 V to 0.4 V.

The cathodic peaks that have been noticed at approximately − 0.5 to −0.01 V on reverse scan are coinciding with ((AZ/BC)/MB reduction as concluded from the reduction peak. Reduction peak intensity drastically reduced at 0.5 M from methanol, in some instances, because of the rise in methanol concentration, because of product inhibition accumulated. The oxidative character of Na₂S₂O₈ also influences this activity by favouring the formation of oxidized species on the surface of the catalyst, potentially favouring more blocking intermediate buildup to redox cycling of active sites^72^. More details on (AZ/BC)/MB behaviour at greater concentrations in a 0.5 M Na₂S₂O₈ electrolyte are accessed through CV plots for 0.37 M and 0.5 M concentrations of methanol. At both these concentrations, an anodic peak indicative of the Methanol Oxidation Reaction (MOR) at about 0.8 V is noticed. For the concentration 0.37 M, the anodic peak current density is about 226.55 mA/cm2, signifying that MOR is a very electrocatalytic process at this concentration^51^.

#### Chronoamperometry and stability analysis

**Fig. 11:**
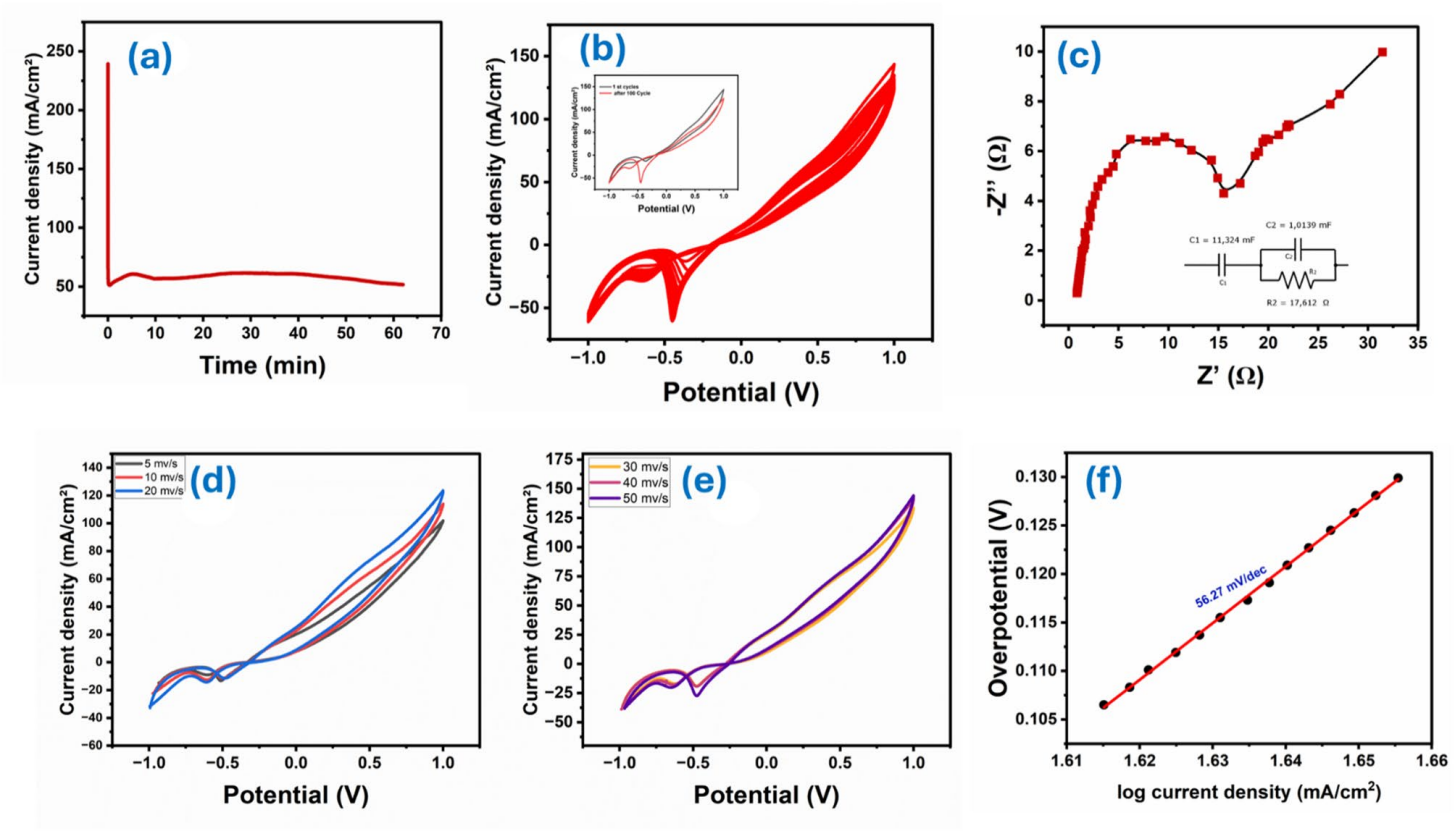
Electrochemical characterization of AZ/BC biomaterial/MB modified electrode, including (**a**) chronoamperometry response, (**b**) CV stability over 100 cyclic, (**c**) EIS impedance Nyquist plot, (**d**) effect of CV scan rate at low scan rate, (**e**) effect of CV scan rate at high scan rate, and (**f**) Tafel polarization plot.

The stability study of sustainability in fuel Cell energy is a fundamental criterion of energy sources. CA measurements were performed to assess the stability of the modified (AZ/BC)/MB dye electrocatalyst in 0.5 M Na_2_S_2_O_8_ electrolyte with 0.37 M methanol for 3600s. The CA response of the (AZ/BC)/MB dye electrode is given in Fig. 11a. It can be noted that the (AZ/BC)/MB electrode gives an outstanding current density of 61 mA/cm2 at 3600s. This indicates the excellent stability and durability of the final modified electrode material, suggesting its potential as an efficient electrocatalyst for DMFCs applications.

Continuous repeating CV cycles (100 cycles) were employed to assess the (AZ/BC)/MB electrode long term cyclic stability. The results are displayed in Fig. 11b and provide information on the electrode material’s susceptibility to the long term electrochemical process^73^. The first cycle, and 100th cycle were presented onset in Fig. 11b. It can be noted that the current density of the anodic oxidation peak reduced to 92.5% after 100th cycle. The declined in current density can be related to the changes in the active sites of the catalyst, which is a general feature of comparable electrocatalyst. This suggests the (AZ/BC) biomaterial electrocatalyst may maintain stability over multiple continuous MOR cycles.

#### Electrochemical impedance spectroscopy (EIS)

Electrochemical impedance spectroscopy (EIS) reflects the charge transfer processes between the active electrode and the electrolyte. Figure 11c presents the Nyquist plots of the (AZ/BC)/MB electrode measured in 0.5 M Na2S2O8 and 0.37 M methanol. Two distinct regions are observed in the Nyquist plot a semicircular portion corresponding to charge transfer resistance (Rct) and double layer capacitance (Qdl), and a linear portion associated with mass transfer processes. These EIS indicate that the system is governed by combined kinetic and diffusion controlled mechanisms. The (AZ/BC)/MB electrode exhibited improved conductivity and reduced equivalent series resistance (R_ct_), as evidenced by the observed semicircular and linear features. Figure 11c onset reveals that (AZ/BC)/MB electrode had low semicircle with a R_ct_ of (17,612 Ω) showing that (AZ/BC)/MB electrode has rapid and effective electron transport. These results illustrate the significance of incorporating the (AZ/BC)/MB which low the electrode resistance and enhances its electrical conductivity.

#### Effect of scan rate

The effect of varying scan rate on the methanol electro-oxidation at the (AZ/BC)/MB electrode was investigated in 0.5 M Na_2_S_2_O_8_ and 0.37 M CH_3_OH using both low (5, 10, 20 mV/s) and high (30, 40, 50 mV/s) scan rates. The results shown in Fig. 11(d-e) indicate that the anodic current density increased steadily with increasing scan rate, rising from 101.98 to 143.40 mA.cm^− 2^ as the scan rate increased from 5 to 50 mV/s. with increasing scan rate, the anodic peak potential (E_p.a._) shifted toward higher (more positive) vales while the cathodic peak potential (E_pc_) moved toward lower (more negative) values. This reveals that there exist diffusion-controlled systems and the electro-oxidation of methanol on catalysts.

#### Tafel polarization plot

The Tafel plot in Fig. 11f demonstrates that the kinetics of the hydrogen evolution reaction (HER) were studied occur in a 0.5 M Na_2_S_2_O_8_ as electrolyte. Tafel slopes are utilized to ascertain values from CV curves by fitting data into the Tafel Eq. (14), which illustrates the relationship between current density and overpotential. The reduced Tafel slope and steady decline in overpotential facilitate an accelerated rate of HER kinetics^74^. The Tafel slopes of 56.27 mV dec⁻¹ confirmed Vollmer, Heyrovsky, and Tafel as the rate-determining step of the process. Tafel slope of (AZ/BC)/MB electrode (56.27 mV/dec) (α = 1.05) is the least, which increases the Volmer process. This results suggest that the modification of electronic structure on the surface of (AZ/BC)/MB accelerates the electron transfer, therefore enhancing the electrocatalytic kinetics^75^.

14 $$\:\eta\:=\alpha\:+b\mathrm{log}j$$

In the following equation, η is the overpotential, α is the Tafel coefficient, b is the Tafel slope, and j is the current density is illustrated Eq. (15)^74^.

15 $$\:b\:=\:\frac{\left(2.303RT\right)}{\left(\alpha\:F\right)\:}\:$$

where R is the universal gas constant (8.314 kJ mol^− 1^ K^− 1^), T is the temperature in Kelvin, α is the charge-transfer coefficient, and F is the Faraday constant (96,485 C mol^− 1^)^74^.

### Computational results

**Fig. 12:**
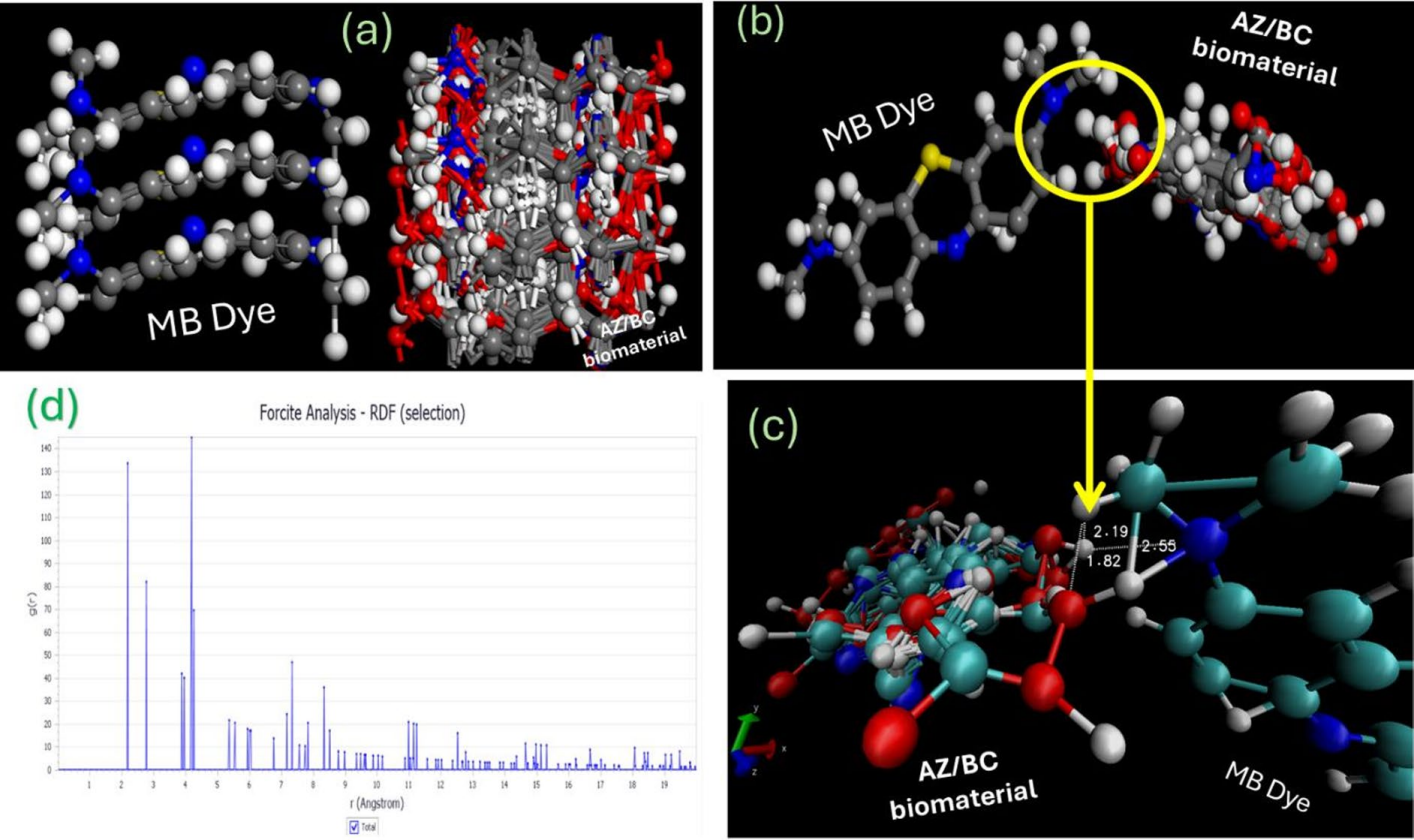
Molecular simulation analysis of MB dye adsorption on AZ/BC biomaterial composite (**a**) representative adsorption configurations obtained Monte Carlo and (**b**) optimized simulation conformation showing the equilibrium structure of the adsorbed MB molecule on the AZ/BC biomaterial composite surface, (**c**) the adsorption geometry highlighting key interaction and bond length (Å) between MB dye and surface atoms, and (**d**) The RDFs for selected interaction sites of MB dye with (AZ/BC) biomaterial surface atoms under dry condition, indicating preferred adsorption distances and interaction strength.

Figure 12(a-c) illustrates the minimal configuration resulting from the adsorption of methylene blue dye onto the (AZ/BC) biomaterial surface in a dry state (without solvent). The Table 4 presents the adsorption (E_ads_), interaction (E_int_), and deformation (E_def_) energies of MB dye absorbed on the (AZ/BC) biomaterial surface, together with the substrate-adsorbate configurations (dEads/dNi), where one component of the adsorbate has been removed. There are various hydrogen bond (HB) donor and acceptor sites in the MB dye molecule. Thus, at a distance of 2.55 Å, a hydrogen bond has been formed between the (AZ/BC) biomaterial’s hydrogen atom and the nitrogen atom of the methylene blue dye molecule.

Furthermore, the (AZ/BC) biomaterial’s oxygen atom has a hydrogen bond with the methylene blue dye molecule’s hydrogen atom at a distance of 1.82 Å and with the hydrogen atoms of the two systems at a distance of 2.19 Å, as illustrated in Fig. 12c. The stability of the adsorbed structure is facilitated by these interactions. The findings of the Eads demonstrate that the methylene blue dye absorbed on the (AZ/BC) biomaterial surface is positive, indicating that the adsorption of the dye is endothermic. Furthermore, it was revealed that the MB dye was absorbed in a parallel pattern on the (AZ/BC) biomaterial’s surface, revealing the strong connections between the methylene blue dye and the surface’s atoms. Analysis of the molecular structures of the methylene blue dye and the surface (physical adsorption) reveals that the electrons of nitrogen and oxygen may have contributed to the MB dye’s adsorption onto the (AZ/BC) biomaterial surface. Furthermore, the Van Der Waals dispersion forces are responsible for the physical adsorption of the methylene blue dye onto the (AZ/BC) biomaterial’s surface, corroborating the results of the experiment. Table 5.

**Table 5:** computational study parameters for the adsorption of MB dye on AZ/BC biomaterial composite.

Total energy (10^3^)	Adsorption energy (10^3^)	Rigid adsorption energy (10^2^)	Deformation energy (10^2^)	MB dE_ad_/dN_i_ (10^3^)
1.271657	1.183770	8.19	3.64	1.183770

The radial distribution function (RDF) was employed to determine the bond length between the atom of MB and the (AZ/BC) biomaterial surface (−1 0 0) analysis of these bond lengths enabled the identification of the types of interactions involved. The bond lengths ranging from 1Å to 3.5Å were typically associated with chemisorption, whereas values greater than 3.5 Å correspond to physisorption interaction. In Fig. 12d was illustrated the RDF fluctuations as a function of bond length (r) between the (AZ/BC) and MB dye is 4.17Å. which indicates that the primary interaction between MB dye and the AZ/BC surface (−1 0 0) is of physisorption type. This funding reflects the strong affinity of MB molecules for adsorption onto the AZ/BC surface.

**Fig. 13:**
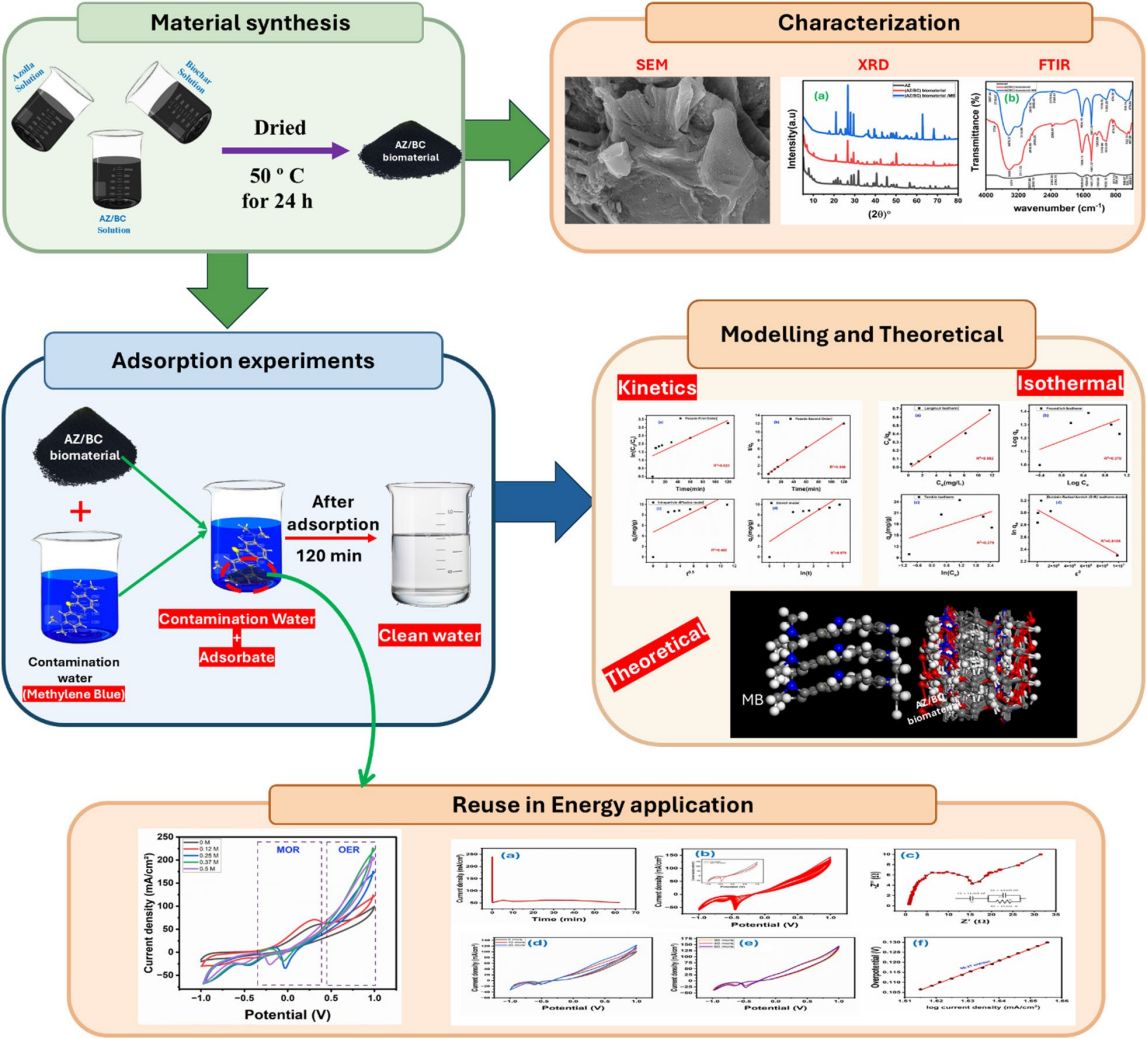
A schematic diagram of the experimental workflow (material synthesis → characterization → adsorption experiments → modelling and theoretical → Reuse biomaterial in energy application).

The combined experimental approach to developing the AZ/BC biomaterial presented in Fig. 13 is an integrated process that combines material synthesis, characterization, adsorption testing, and theoretical modelling, thereby facilitating re-use of the AZ/BC biomaterial for sustainable energy applications. Table 6.

**Table 6:** Comparative literature summary of adsorption performance for MB and other pollutants using various adsorbents.

Catalyst	Pollutant	Parameters	Efficiency	Ref.
Clinoptilolite/Fe3O4 (Clin/Fe3O4)	MB	initial pH of 10.	(97.57%)	^76^
Alginate/Clinoptilolite/Fe3O4 (Alg/Clin/Fe3O4)	MB	initial pH of 10.	(93.62%)	^76^
he pyrolysis of mint stalks and lemon peels was performed to synthesize mint-stalks (MBC) and lemon-peels (LBC)	MB	within 90 min	87.5% and 60% Respectively	^20^
Na-bentonite	MB	120 rpm agitation speed, 60 min contact time, 4 gL − 1 adsorbent dose and 30 °C	90%	^77^
activated carbon/natural zeolite (AC/NZ)	MB	45 min	99.2%	^18^
Rough reed fibers (R)R and Treated reed fibers (RS)	MB	at pH = 5.	69.62% and 91.09% respectively	^17^
almond shell modified with NaOH (AMAS)	MB	0.1 M of HNO3 solution	98.29%	^78^
Giant reed biochar	Basic blue 41 (BB41) and Eriochrome black T (EBT)	-	98.6% and 82.5% respectively	^79^
pyrolyzed Zeolite-Biochar Composite (PZC 7:3)	Phosphate	-	90%	^80^
modified wood biochar	doxycycline and ciprofloxacin,	-	96.23% and 96.90% respectively	^81^
aluminum oxide nanoparticles (AONPs)	MB	6 (pH), 200 (rpm), and 30 (minutes)	97.34%	^82^
modified rich silica sand (MRSS), enhanced with KOH	amoxicillin (AMO)	(pH 3, stirring speed 200 rpm, contact time 3 h)	94.6%	^83^
silica sand coated with synthesized aluminum oxide nanoparticles (SSC–Al2O3)	MB	At the ideal pH (6), under an agitation speed of 200 rpm and 75 min	95.33%	^84^
AZ/BC	MB	Within 120 min	96.21%	This work

### Conclusion

The objective of this study was to reuse of adsorption wastes in the electrooxidation of methanol. the adsorption of Methylene blue (MB) using a (AZ/BC) biomaterial made of biochar and Azolla extract. SEM indicated a dramatic modification of the Azolla extract surface, going from a coarse texture with many variably sized pores to a regularly layered structure after the creation of the (AZ/BC) biomaterial. the maximum removal efficiency of (AZ/BC) biomaterial for MB adsorption was 96% and an adsorption capacity of 10 mg g⁻¹ for a duration of 120 min. The experimental adsorption data exhibited a strong correlation with the Langmuir linear isotherm model. chronoamperometry and the Cyclic Stability verified. The stability of bioelectrode the (AZ/BC) biomaterial electrode showed outstanding stability for the length of the 60-minute chronoamperometry test and for 100 cycles in the Cyclic Stability experiment. This approach accomplishes environmental balance through recyclable biocatalysts for energy applications and gives an opportunity to valorize waste materials as effective electrocatalystsFinally, the adsorption of MB on the AZ/BC biomaterial was further investigated using the Monte Carlo (MC) by adsorption locator module which confirmed a strong and highly reactive interaction between AZ/BC biomaterial surface and MB dye. In addition MC simulations were conducted to evaluate the adsorption behaviour of the MB dye on the AZ/BC biomaterial surface.

## Data Availability

The Data are analyzed during the current study are available from the corresponding author on request.
